# Assessment of the Hawthorne effect during central venous catheter manipulation

**DOI:** 10.1590/1980-220X-REEUSP-2022-0125en

**Published:** 2022-09-02

**Authors:** Renata Desordi Lobo, Maura Salaroli de Oliveira, Juliana Jorge Colella, Natalia Dalforno da Silva, Laerte Pastore, Regina Claudia da Silva Souza

**Affiliations:** 1 Hospital Sírio Libanês, Serviço de Controle de Infecção Hospitalar, São Paulo, SP, Brazil.; 2 Hospital Sírio Libanês, Unidade de Terapia Intensiva, São Paulo, SP, Brazil.; 3 Hospital Sírio Libanês, Núcleo de Novos Conhecimentos, São Paulo, SP, Brazil.

**Keywords:** Central Venous Catheters, Catheter-Related Infections, Hand Hygiene, Evidence-Based Practice, Nursing, Team, Cateteres Venosos Centrais, Infecções Relacionadas a Cateter, Higiene das Mãos, Prática Clínica Baseada em Evidências, Equipe de Enfermagem, Catéteres Venosos Centrales, Infecciones Relacionadas con Catéteres, Higiene de las Manos, Práctica Clínica Basada en la Evidencia, Grupo de Enfermería

## Abstract

**Objectives:**

To describe the compliance to the practices of hand hygiene and hub disinfection before manipulation of the central venous catheter in two moments: before and after educational intervention. Adherence to hand hygiene was assessed with two methods: direct observation and video camera.

**Methods:**

Before and after study conducted with the nursing team in an adult intensive care unit, in São Paulo, Brazil, including 180 observations using video cameras and direct observation. Hand hygiene compliance before catheter manipulation and compliance with the correct technique and the hub disinfection for five seconds were observed.

**Results:**

When video cameras recording was observed, hand hygiene compliance increased from 46% to 66% and the use of the proper technique increased from 23% to 46% (p < 0.05). Regarding hub disinfection compliance, no difference was observed between the periods. Hand hygiene compliance in direct observation increased from 83% to 87% and in indirect observation, from 46% to 66% after the intervention.

**Conclusion:**

After the educational intervention, hand hygiene compliance before CVC manipulation and the use of the correct technique improved. When observed indirectly, the adherence to these practices was lower, reinforcing the Hawthorne effect.

## RESUMO

**Objetivos:**

Descrever o cumprimento das práticas de higienização das mãos e desinfecção dos conectores antes da manipulação do cateter venoso central antes e após intervenção educativa. A adesão à higienização das mãos foi avaliada por observação direta e câmera de vídeo.

**Métodos:**

Estudo antes e depois realizado com a equipe de enfermagem em unidade de terapia intensiva para adultos, em São Paulo, Brasil, com 180 observações com câmeras de vídeo e observação direta. A adesão à higienização das mãos antes da manipulação do cateter e à técnica correta e desinfecção do conector por cinco segundos foram observadas.

**Resultados:**

Quando as câmeras de vídeo foram observadas, a adesão à higiene das mãos aumentou de 46% para 66% e o uso da técnica adequada aumentou de 23% para 46% (p < 0,05). Em relação à adesão à desinfecção do conector, não foi observada diferença entre os períodos. A adesão à higienização das mãos na observação direta aumentou de 83% para 87% e na indireta, de 46% para 66% após a intervenção.

**Conclusão:**

Após a intervenção educativa, houve melhora na adesão à higienização das mãos antes da manipulação do cateter venoso central e no uso da técnica correta. Quando observada indiretamente, a adesão a essas práticas foi menor, reforçando o efeito Hawthorne.

## RESUMEN

**Objetivo:**

Describir el cumplimiento de las prácticas de higiene de manos y desinfección del *hub* antes de la manipulación del catéter venoso central en dos momentos: antes y después de la intervención educativa. La adherencia a la higiene de manos se evaluó con dos métodos: observación directa y cámara de video.

**Métodos:**

Estudio antes-después realizado con el equipo de enfermería en una unidad de cuidados intensivos de adultos, en São Paulo, Brasil, que incluye 180 observaciones utilizando cámaras de video y observación directa. Se observaron el cumplimiento de la higiene de manos antes de la manipulación del catéter y el cumplimiento de la técnica correcta y la desinfección del *hub* durante cinco segundos.

**Resultados:**

Cuando se observó la grabación de cámaras de video, el cumplimiento de la higiene de manos aumentó del 46% al 66% y el uso de la técnica adecuada aumentó del 23% al 46% (p<0,05). En cuanto al cumplimiento de la desinfección de *hubs*, no se observó diferencia entre los períodos. El cumplimiento de la higiene de manos en observación directa aumentó del 83% al 87% y en observación indirecta del 46% al 66% tras la intervención.

**Conclusión:**

Después de la intervención educativa mejoró el cumplimiento de la higiene de manos antes de la manipulación del CVC y el uso de la técnica correcta. Cuando se observó indirectamente, la adherencia a estas prácticas fue menor, reforzando el efecto Hawthorne.

## INTRODUCTION

Patients admitted to Intensive Care Units (ICUs) have severe and acute conditions, and a central venous catheter (CVC) is widely used in this scenario. Approximately 90% of patients in these units have an intravascular catheter, of which 60% were non-tunneled catheters^(1)^. Despite the countless benefits and possibilities provided by CVC, its use is associated with possible complications, the most common being bloodstream infection (BSI), venous thrombosis, and catheter obstruction or malfunction^(2,3,4)^. Thus, to avoid adverse events, proper management of central venous catheters by medical and nursing teams is essential^(5)^.

Guidelines recommend periodic education of health professionals on catheter insertion and manipulation practices, as well as periodic assessment of their knowledge and adherence to these practices. The CVC bundle for infection prevention has five key components: hand hygiene; maximal barrier precautions; chlorhexidine skin antisepsis; optimal catheter site selection, with avoidance of the femoral vein for central venous access in adult patients; and daily review of line necessity, with prompt removal of unnecessary lines^(1,6,7)^.

One of the most important factors is daily catheter care after insertion. In this sense, hand hygiene before and after accessing intravascular catheter and hub disinfection are essential measures to prevent bloodstream infection. A set of indicators related to a specific action may be necessary to describe the quality provided in a process. This measure is important to determine if the actions and steps recommended in a practice are being followed^(8)^. These process indicators will be the main parameters to be used to measure the quality of care, as they do not consider the result, but the actions necessary to achieve it, showing the exact point that can be improved by the professional to achieve better outcomes^(6–9)^.

There are many examples of Evidence Based Practice (EBP) in the nursing daily practice and nurses play a key role in helping to prevent illness before it happens by adhering to evidence-based infection-control policies. However, we should consider how to audit adherence to best practices. It is unclear how the Hawthorne effect influences the care provided by health professionals^(10)^ and its impact on clinical practice. In a systematic review conducted on the Hawthorne effect in professionals, approximately 78% studies showed behavioral changes among health professionals when they were observed. In all studies, the behavior was “positive,” that is, characterized by changes such as increased productivity, compliance, or adherence to the guideline or best practice protocol^(10)^.

In general, healthcare personnel have low rates of compliance despite widespread and longstanding recognition that hand hygiene adherence is the required strategy to reduce hospital infection rates^(11,12)^.

Hand hygiene (HH) is one of the main actions observed and demanded from healthcare professionals. Monitoring can be accomplished using several different methods, though the gold standard is direct observation of health care provider practices by a trained observer^(13,14,15)^. However, this practice has some limitations and biases, the main one being the influence of an observer’s presence, since it produces the Hawthorne effect^(16,17)^. The Hawthorne effect in this practice has been extensively studied because it implies different hand hygiene compliance when direct and presential observation and indirect observation are compared, that is, when the workers do not know that they are being observed and evaluated^(16)^. The Hawthorne effect was clearly seen in the increase in HH compliance among health professionals observed by trained personnel^(18)^. Similarly, a comparison of HH compliance by doctors and nurses between direct and indirect observations showed that the compliance rate was higher when the observer was present than when the observer was absent^(17)^.

There are several limitations to using direct observation for hand hygiene monitoring. Direct observation is time consuming and compliance data may be influenced by the Hawthorne effect, in which providers change behavior when they are aware of the presence of an observer. The use of different methods, like video surveillance for compliance had been observed in many different locations, such as football (https://football-technology.fifa.com/media/172213/handbook-virtual-offside-line-assessment.pdf). It is also used in hospital settings too for different purposes. Some studies have used video monitoring, such as for falling, medication errors, and hand hygiene monitoring as well^(19)^.

The indirect observation of the professional with the use of video cameras may minimize the Hawthorne effect, measuring the real adherence to the practices. This strategy can be used to identify reliable indicators of adherence to best practices by healthcare professionals and contribute to ascertaining the actual team’s educational needs and to implementing improvements in outcomes.

The objectives of study were to evaluate compliance with hand hygiene and hub disinfection before CVC manipulation in two moments, before and after education intervention, using video cameras, and to compare compliance hand hygiene using two methods, direct observation and video cameras.

## METHOD

### Type of Study

Before and after study.

### Setting

This study was conducted at private, JCI accredited, tertiary hospital with 450 beds and 28 general ICU beds, from July 2018 to December 2019, divided into 4 subperiods:

– Baseline period (July 2018 to June 2019)
– Pre-intervention period (June 2019 to July 2019)
– Intervention period (August 2019)
– Post-intervention period (September 2018 to December 2019)

### Data Collection

During the **baseline period**, CVC-BSI rates were prospectively evaluated, as part of infection control routine, based on NHNS criteria^(20)^.

During the **pre-intervention period**, compliance hand hygiene and hub disinfection were assessed through video camera observations (indirect observations). Thus, the professionals had no information on when they were being observed. The 90 observations were conducted at random and with a maximum limit of five observations per professional per day to avoid the bias of repeating the same appropriate or inappropriate behavior and were within the morning, afternoon, and night shifts, totaling 30 observations for each period. We observed the practices of hand hygiene compliance before catheter manipulation and hub disinfection before administration of medications.

The **intervention period** consisted of education and training tailored to directed problems found during the observation phase. The activity included 24 training sessions of 20 to 30 minutes each, grouping an average of five professionals per session totaling 110 professionals, 77% from staff. The sessions were held during the working hours of all shifts in a training room inside the studied unit. The intervention was an educational reflective activity with the presentation of an expository content on BSI rates in the unit in recent years and the most recent CVC-BSI cases. The best practices in CVC manipulation described in the literature were also addressed, emphasizing the points with less adherence by professionals that were identified in the first observation. At the end of the training, there was a debriefing: four multiple-choice questions on the topic were presented, so that the professionals could reflect and discuss among themselves, which the best answer was, and at the end of each question, the mediator confirmed the correct alternative and the group reflected about it.

**Post-intervention period**, the observation of compliance with hand hygiene and hub disinfection was performed through video cameras.

At all stages, evaluation of hand hygiene compliance from direct observation was made.

## THE DEFINITIONS USED FOR THIS STUDY WERE

– Hand hygiene compliance was defined as health care workers (HCW) that used alcohol or soap and water immediately before CVC manipulation (moment 2 – WHO Hand Hygiene) and correct handwashing technique with soap and water or hand disinfection with hand sanitizer according to the techniques recommended by the World Health Organization. Palm full of the product in a cupped hand and covering all surfaces; rub hands palm to palm; right palm over left dorsum with interlaced fingers and vice versa; palm to palm with fingers interlaced; backs of fingers to opposing palms with fingers interlocked; rotational rubbing of left thumb clasped in right palm and vice versa; rotational rubbing, backwards and forwards with clasped fingers of right hand in left palm for 40 to 60 seconds^(21)^.
– Hub disinfection compliance, use a pad device with 70% alcohol to disinfect catheter hub and correct technique for hub disinfection with 70% alcohol for 5 seconds.

### Setting

The study was conducted in a private, JCI accredited, tertiary care hospital, which has 548 beds (95 are ICU beds) and approximately 6,000 employees in São Paulo, Brazil. The general ICU which has 28 beds and a team of 46 nurses and 97 nursing technicians (nursing team). Each bed already had a videocamera for security surveillance.

### Data Collection

The instrument used for data collection was a form including sociodemographic variables such as sex, professional category, and work shift, and variables related to catheter manipulation, such as hand hygiene immediately before CVC manipulation, correct HH technique, hub disinfection for five seconds before catheter manipulation.

In Direct observations, six trained observers responsible for assessing hand hygiene compliance on the five HH moments. They are nursing technicians distributed in different shifts. The number of observations evaluated was the same before and after intervention education.

### Data Analysis and Processing

Information was stored in a Microsoft*®* Office Excel 2018 spreadsheet and the statistical analyses were performed using the Open Epi software version 3.01. Analysis to compare compliance to HH and CVC manipulation in the periods before and after the intervention through video cameras was performed using the Chi-square test with Yates correction or the Fisher’s Exact test.

### Ethical Aspects

The project was approved by the Ethics Committee of the hospital on November 14, 2019, and conducted in accordance with Resolution 466, of December 2012, registry number 3.706.807.

## RESULTS

This study collected data on 180 CVC manipulation observations, 90 before and 90 after the intervention. Regarding the sociodemographic characteristics of the professionals observed, 176 (98%) were nursing technicians and 76% were female.

The educational intervention reached 135 (77%) of the nursing professionals in ICU.

Table 1 shows the rates of compliance before and after the educational intervention. There was an improvement of HH compliance and the practice of correct technique.

**Table 1.** Rates of hand hygiene compliance and hub disinfection observed using video cameras in the periods before and after the educational intervention – São Paulo, SP, Brazil, 2019.

Variable	Before intervention	After intervention	P^*^
	n (90)	n (90)	
HH compliance before CVC manipulation	41 (46%)	59 (66%)	0.006
HH (correct technique)	21 (23%)	41 (46%)	0.002
Hub disinfection compliance	85 (94%)	86 (96%)	0.732
Hub disinfection (correct technique)	49 (54%)	50 (56%)	0.881

HH: Hand Hygiene. *Chi square test.

Regarding the comparison of compliance of HH evaluated through direct observation and video cameras observation, we noticed that, during the pre-intervention period, hand hygiene compliance through direct observation was 64/77 (83%), compared to 46% through video cameras (41/90). In the post-intervention period, HH compliance through direct observation was 87% (81/93) and through video cameras 66% (59/90). There was no difference between work shifts, as described in Table 2.

**Table 2.** Compliance of HH evaluated by direct observation and by video cameras before CVC manipulation – São Paulo, SP, Brazil, 2019.

Variable	Before intervention	After intervention	P^*^
HH direct observation	64/77 (83%)	81/93 (87%)	0.465
HH video cameras observation	41/90 (46%)	59/90 (66%)	0.006

HH: Hand Hygiene. *Chi square test.

From July 2018 through June 2019, a total of 4 cases of CVC BSI occurred, corresponding to a rate of 0.78 episodes per 1000 CVC-days. From July 2019 through December 2019, one case of CVC BSI, corresponding to a rate of 0.32 cases per 1000 CVC-days.

## DISCUSSION

This study examines the real-world practices of monitoring and implementing the Central Line Bundle in ICU. Adherence to hand hygiene, as well as the correct hand hygiene technique, was one of the relevant points after the implementation of the education strategy on good practices in CVC manipulation. Even with a raise of HH compliance and correct technique, we consider further efforts to increase compliance as our goal is always 100%, especially before handling the CVC to prevent bloodstream infection. Another interesting result was that the increase in hand hygiene practices compliance had an impact depending on the form of audit performed, with lower rates when indirect observation was used.

Data related to the characterization of the professionals participating in the study corroborate the 85% predominance of female nursing professionals, reported in 2017 by the Brazilian Federal Nursing Council, which records the Nursing Profile in Brazil^(22)^. This shows a predominance of females and of nursing technician professionals. An aspect that deserves attention is that in Brazil, the professionals who most administer medication through central venous catheters are the nursing technicians, a fact observed in this study and in other studies with similar objectives^(4)^.

A continuous education project is necessary to effectively decrease infection rates, understanding that specific interventions can only produce momentary improvements. The professionals observed demonstrated a high level of theoretical knowledge about CVC care practices. However, as the literature demonstrates, the greatest difficulty is to keep them continuously committed to performing these practices, with systematic and continuous training programs being required^(23)^.

The comparison of hand hygiene compliance between the present study and other studies that measured adherence to this practice by indirect observation shows that the adherence in this study is similar to the mean. As healthcare professionals can often neglect infection control practices, video camera monitoring can be an intervention associated with other important actions in infection prevention and control^(23)^. Some factors can contribute to better adherence to health practices, such as adequate infrastructure and access to devices and materials for the procedure, such as the distribution of alcohol dispensers at each bed.

The mean hand hygiene compliance among health professionals is 40%. However, direct observation is the most frequent method used in measuring this adherence in studies, which is described as an important bias due to the Hawthorne effect. In the present study, this variable should not be considered since the observation was performed using cameras and demonstrated that when observed by video cameras, professionals have less adherence to hand hygiene practices before handling the central venous catheter compared to direct observation^(24)^.

Even with training, the correct HH technique is a common problem in several scenarios. A study conducted with 1269 professionals showed that 33% of them performed the procedure incorrectly^(25)^. The results of present study show an even greater deficit regarding the technique, in which 23% to 46% of the observed HH were not performed with the correct technique, reinforcing the deficiency and showing the need to improve this practice.

The compliance rates to hub disinfection observed in the present study are high (more than 90%). However, when considering the time of five seconds for this practice, only 56% of the procedures were performed properly, even after the intervention.

The results of this study show the influence of the presence of the observer, reinforcing the existence of the so-called Hawthorne effect, described in the literature^(10,16)^. The rates of hand hygiene compliance with direct observation in the period before and after the intervention were above the rates with indirect observation. This suggests that the presence of the auditor on the scene interferes with the action of the professionals at that moment, as it can influence them to remember the correct procedure, resulting in a “false adherence”.

One aspect that supports this theory is the difference between the periods before and after the educational intervention, comparing the two observation methods. Direct observation resulted in increased adherence to practices in both moments of the study, suggesting that professionals performed hand hygiene before handling the CVC in two periods, suggesting the influence of the auditor’s presence. The difference in compliance between the periods before and after, with observation by cameras, showed a smaller increase in compliance. Although the adherence rates are different, the increase related to the indirect observation technique is more reliable, because the professionals were able to remember and carry out this practice regardless of the presence of third parties at the site.

We suggest that the video observations complement the direct observations to the service that has this resource. Despite the Hawthorne effect, direct observation itself appears to be an intervention as it allows for immediate intervention. This is a good topic for discussion.

Our study has limitations. For instance, the educational intervention was not carried out with all professionals.

## CONCLUSION

This study showed a difference in the rate of hand hygiene compliance between direct and indirect observation (using video cameras), with lower compliance rates in indirect observation, which reinforces the influence of the observer’s presence. This aspect encourages the method of indirect observation when evaluating compliance to a determined care practice, since the obtained data are more reliable and real. It is necessary to invest in research that continuously monitors the professionals’ compliance to best practices, especially those related to infection prevention. Having a written CL Bundle policy in place is no guarantee they are followed. Continuous rigorous monitoring of compliance with good technique in using it may be required.

## References

[1] O’Grady NP, Alexander M, Burns LA, Dellinger EP, Garland J, Heard SO (2011 May). Guidelines for the prevention of intravascular catheter-related infections. Clin Infect Dis..

[2] Chopra V, Anand S, Krein SL, Chenoweth C, Saint S (2012 Aug). Bloodstream infection, venous thrombosis, and peripherally inserted central catheters: reappraising the evidence. Am J Med..

[3] Pikwer A, Åkeson J, Lindgren S (2012 Jan). Complications associated with peripheral or central routes for central venous cannulation. Anaesthesia..

[4] Silva JAJ, Ferreira LA, Zuffi FB, Rezende MP, Mendonca GS (2018 May/June). Breakdown of complications related to the use of central venous catheters in intensive therapy units. Biosci J.

[5] Safdar N, Maki DG (2004 Jan). The pathogenesis of catheter-related bloodstream infection with noncuffed short-term central venous catheters. Intensive Care Med..

[6] Institute for Healthcare Improvement (2012). How-to guide: prevent central line-associated bloodstream infection [Internet].

[7] Brasil. Agência Nacional de Vigilância Sanitária (2017). Medidas de prevenção de infecção relacionada à assistência à saúde [Internet].

[8] São Paulo. Secretaria de Estado da Saúde. Divisão de Infecção Hospitalar (2006). Centro de Vigilância Epidemiológica. Manual de avaliação da qualidade de práticas de controle de infecção hospitalar [Internet].

[9] Campbell SM, Braspenning J, Hutchinson A, Marshall MN (2003 Apr). Research methods used in developing and applying quality indicators in primary care. BMJ..

[10] McCambridge J, Witton J, Elbourne DR (2014 Mar). Systematic review of the Hawthorne effect: new concepts are needed to study research participation effect. J Clin Epidemiol..

[11] Larson EL, Early E, Cloonan P, Sugrue S, Parides M (2000). An organizational climate intervention associated with increased handwashing and decreased nosocomial infections. Behav Med..

[12] Pittet D, Hugonnet S, Harbarth S, Mourouga P, Sauvan V, Touveneau S (2000 Oct). Effectiveness of a hospital-wide programme to improve compliance with hand hygiene. Infection Control Programme. Lancet..

[13] World Health Organization (2009). Guidelines on hand hygiene in health care: first global patient safety challenge clean care is safer care [Internet].

[14] Marschall J, Mermel LA, Fakih M, Hadaway L, Kallen A, O’Grady NP (2014 Jul). Strategies to prevent central line-associated bloodstream infections in acute care hospitals: 2014 update. Infect Control Hosp Epidemiol..

[15] World Health Organization (2009). Formulating strategies for hand hygiene promotion. In: World Health Organization. WHO Guidelines on Hand Hygiene in Health Care: First Global Patient Safety Challenge Clean Care is Safer Care [Internet].

[16] Wu KS, Lee SS, Chen JK, Chen YS, Tsai HC, Chen YJ (2018 Aug). Identifying heterogeneity in the Hawthorne effect on hand hygiene observation: a cohort study of overtly and covertly observed results. BMC Infect Dis..

[17] Kovacs-Litman A, Wong K, Shojania KG, Callery S, Vearncombe M, Leis JA (2016 Dec). Do physicians clean their hands? Insights from a covert observational study. J Hosp Med..

[18] Gould D, Lindström H, Purssell E, Wigglesworth N (2020 Jul). Electronic hand hygiene monitoring: accuracy, impact on the Hawthorne effect and efficiency. J Infect Prev..

[19] Armellino D, Hussain E, Schilling ME, Senicola W, Eichorn A, Dlugacz Y (2012 Jan). Using high-technology to enforce low-technology safety measures: the use of third-party remote video auditing and real-time feedback in healthcare. Clin Infect Dis..

[20] National Healthcare Safety Network (NHSN) (2022). Bloodstream Infection Event (Central line-associated bloodstream infection and non-central line associated bloodstream infection) [Internet].

[21] World Health Organization (2006). How to handrub? How to handwash? [Internet].

[22] Conselho Federal de Enfermagem (2016). Pesquisa perfil da enfermagem no Brasil [Internet].

[23] Karabay M, Kaya G, Hafizoglu T, Karabay O (2019 Nov). Effect of camera monitoring and feedback along with training on hospital infection rate in a neonatal intensive care unit. Ann Clin Microbiol Antimicrob..

[24] Bolon MK (2016 Sep). Hand hygiene: an update. Infect Dis Clin North Am..

[25] Lehotsky Á, Morvai J, Szilágyi L, Bánsághi S, Benkó A, Haidegger T (2017 Jul). Hand hygiene technique assessment using electronic equipment in 26 Hungarian healthcare institutions. Orv Hetil..

